# A comprehensive analysis for wind turbine transformer and its limits in the dissolved gas evaluation

**DOI:** 10.1016/j.heliyon.2024.e39449

**Published:** 2024-10-16

**Authors:** Ricardo Manuel Arias Velásquez

**Affiliations:** Universidad Tecnológica Del Perú, Peru

**Keywords:** Dissolved gas analysis, Fault, Transformers, Wind park

## Abstract

This study employs the PRISMA-A methodology to conduct a systematic review of transformer fault diagnostics using Dissolved Gas Analysis (DGA) data. A comprehensive analysis was performed across four major databases—IEEE, Scopus, ScienceDirect (Elsevier), and Web of Science—yielding 12,511 initial records. Following rigorous evaluation, including duplicate removal and eligibility criteria assessment, 1190 articles underwent statistical evaluation. The search strategy focused on keywords related to transformer faults and diagnostic methods, resulting in a refined dataset of 4810 DGA samples from wind park transformers. Detailed statistical analysis of gas concentrations—hydrogen, methane, carbon monoxide, carbon dioxide, ethylene, ethane, acetylene, oxygen, and nitrogen—revealed significant insights into fault indicators and distribution patterns. Furthermore, predictive modeling using various machine learning algorithms highlighted the efficacy of models such as Random Forest and CART, achieving accuracies up to 95.29 % in fault prediction tasks. Proposed revisions to IEEE gas concentration thresholds aim to enhance early fault detection capabilities, thereby improving maintenance planning and transformer reliability. The findings underscore the importance of advanced analytics and sustainable practices in transformer diagnostics, calling for continued research in predictive maintenance and eco-friendly insulation technologies to meet future energy challenges.

## Highlights

• PRISMA-A analyzed 12,511 records across four database with 4810 samples.
• Machine learning algorithms, including Random Forest and CART, achieved an accuracy of 95.29 %.
• Revised gas concentration thresholds aim to improve early fault detection capabilities.
• The database from the faults in wind parks proposed new limits for the international standards.

## 1 Introduction

The integration of renewable energy, particularly wind power, into the grid presents significant challenges, especially in terms of grid connection and the cost of substations. A critical issue is the underutilization of wind farm transformers, given that the capacity factor of onshore wind turbines is often only around 30 %; and the new projects in the transmission system is belong the average of the quantity of the new wind parks project, a problem detected during 2017–2023. In 2024, a new paper investigated the application of Dynamic Transformer Rating (DTR) to a wind farm expanded to 150 % of its original capacity, connecting to the grid through the same transformer, simulating a 1:1.5 ratio. The primary focus is on forecasting wind farm generation and transformer capacity 36 h ahead to assess operational challenges in day-ahead dispatch planning. The combined model predicts overheating events with a recall of 84.2 % and a precision of 76.1 % for the 110 °C hot spot temperature (HST) limit, and higher accuracy for the 140 °C limit with a recall of 96.2 % and precision of 95.7 %. Despite good performance, the model falsely predicts overheating in some cases, highlighting a need for improved forecasting, especially for wind power, to reduce unnecessary curtailment and enhance transformer operation safety [1].

In 2012, Reference [2] introduced a novel Power Transformer Fault Diagnosis System (PTFDS) designed to enhance the reliability of transformer operation in wind farms. This system incorporated thermal, magnetic, and gas sensors that feed data to a central unit for digital signal conversion and fault type determination. Registered as a patent in Iran, the PTFDS detected faults within milliseconds, showcasing its rapid response and accuracy in fault diagnosis. The experiments also highlighted instances where false detection by one transformer's PTFDS did not affect the accurate detection by the other, underscoring the system's robustness. Thus, the proposed PTFDS represents a significant advancement in transformer fault diagnosis technology, promising enhanced reliability and reduced downtime in wind farm operations [2].

On the other hand, an improvement of the 3D finite element analysis to simulate radial deformations in transformer windings and spectrum frequency response analysis (SFRA) in 2015. By incorporating variations in capacitance and inductance elements, the study demonstrates that even slight radial deformations can be detected through FRA signatures, contrary to earlier assumptions. The key findings include the development of charts correlating the percentage change in electrical parameters with different fault levels, facilitating precise fault simulation using the transformer equivalent circuit model. The research emphasizes that these parameter changes are consistent regardless of fault location, enhancing the robustness of fault detection methodologies. Overall, the proposed methodology enhances understanding and quantification of transformer winding deformations through FRA, offering a reliable framework for detecting and assessing faults critical to transformer health and operational reliability [3].

Besides, in 2020 Doble company detected limits in the daily gasification process for thermal or partial discharge fault, as follows [4].

- More than 100 ppm/day is the most critical condition.
- Between 3 ppm/day to 99.9 ppm/day is a risk condition.
- From 1 ppm/day to 3 ppm/day is a degradation with a failure mode active.
- Lower than 1 ppm/day is a normal condition.

For instance, with internal arcs, this condition should be evaluated and investigated with additional task, and it depends since two points at least.

In 2019, the IEEE developed the international standard for the dissolved gas analysis (DGA), to improve the analysis for transformers regarding to the mineral oil with limitations regarding to renewable energy transformers, especially for wind parks [5]. During 2022, the DGA fault severity interpretation by utilizing screening test statistics and optimization curves with transformer failure data. The study addresses the limitation in IEEE C57.104–2019, which lacks a clearly defined status code higher than 3, by introducing an "Extreme DGA" category. By multiplying the status code 3 limits by a factor of seven, the predictive performance for near-term transformer failure is significantly improved. Screening test statistics, such as the diagnostic odds ratio (DOR) and positive predictive value (PPV), and optimization curves, including the ROC and Precision-Recall (P-R) curves, were used to evaluate the effectiveness of various multipliers. Results indicate that a multiplier of 7 optimizes classifier efficiency and DOR. However, despite this improvement, the IEEE method still falls short compared to PFS (IEC 60599–2015/CIGRE TB 771) and Reliability-based DGA in terms of high-risk case detection. The findings suggest that while the IEEE C57.104–2019 can be considerably enhanced with a simplistic limit multiplier, further algorithmic adjustments using comprehensive failure data are necessary to reach the performance levels of other advanced DGA interpretation methods [6].

### 1.1 Motivation

When applying DGA to wind turbine transformers, it is essential to account for the specific internal components and operational conditions. The elevated gas values often seen in these transformers necessitate a cautious and well-informed approach to DGA interpretation to avoid misdiagnosis of the transformer's health. An internal influence is about the wind turbine transformers frequently incorporate internal auxiliary components, such as load break switches, immersed in the same oil as the transformer. These components' normal operation generates electrical arcs and combustible gases. Quantitative analysis has shown that these auxiliary components can substantially influence DGA results. Impact of Switch Operation:

The gases produced by load break switches during their operation can overshadow the gases generated by the transformer, often leading to measurements that are disproportionately high. Data indicates that gas concentrations from these switches can exceed the typical transformer's gas production by an order of magnitude. This can mask critical issues within the transformer that DGA is intended to reveal, thus complicating maintenance and operational decisions. About the elevated values, the initial data analysis of wind turbine transformer DGA results frequently shows gas values that are significantly elevated—sometimes up to two orders of magnitude higher than those seen in other types of transformers, with a range of 10 ppm, wind turbine transformers might show levels as high as 1000 ppm, therefore, the wind turbine transformers are subject to unique operating conditions, including frequent and wide load fluctuations and close integration with power electronics. These conditions lead to gas generation patterns that differ markedly from those in more traditional transformers. Quantitative studies have revealed that earlier generation wind turbine transformers, which were not designed to withstand such rigorous conditions, exhibit higher gas values. This necessitates a comprehensive understanding of the operating environment to accurately interpret DGA results. Finally, about the complexities associated with wind turbine transformer operation, the interpretation of DGA results should be handled by trained and experienced personnel. A thorough analysis should encompass the overall operating context, transformer nameplate data, factory test reports, operational and maintenance records, and environmental conditions. Quantitative tools such as spreadsheets, databases, analytical software, and maintenance management systems are crucial for accurately assessing transformer health. For example, statistical analysis and trend evaluation using historical data can help differentiate between normal operational gas levels and those indicative of potential issues, it a constraint due to the standards like ASTM D923, the gas concentration measurements should be conducted by analytical laboratories using ASTM D3612 methods or by trained personnel using portable instruments or online monitoring devices. Ensuring consistency in sampling and measurement processes is vital for obtaining reliable and comparable data, which in turn facilitates the accurate interpretation of DGA results [5].

### 1.2 Objective

The objective of this paper is to develop a comprehensive framework for the accurate interpretation of Dissolved Gas Analysis (DGA) results in wind turbine transformers. This framework proposed the significantly influence gas generation patterns. Specifically, the study aims to: Identify and Quantify Internal Influences with its limits in the DGA results, and quantify their contribution to overall gas values, besides, the differentiate Gas Sources, with the gases produced by auxiliary components and those generated by the transformer itself to prevent misdiagnosis of transformer health; and address Elevated Gas Values with the values in wind turbine transformers compared to other transformer types, and develop guidelines for interpreting these elevated values accurately.

The paper has the following content, as follows: Section 2 introduced the methodology with preferred reporting items for systematic reviews and meta-analyses for abstracts (PRISMA-A), with the quantitative and qualitative evaluation, risk bias, and evaluation of the content. Besides, Section 3, describes the results in the wind turbine transformer and its limits for the evaluation of the DGA, with the faults. As a complementary, section 4 introduced the discussion between researchers, international standards and the manufacturers, in the last section the conclusions and future recommendations.

## 2 Methodology

### 2.1 PRISMA-A methodology

**Fig. 1:**
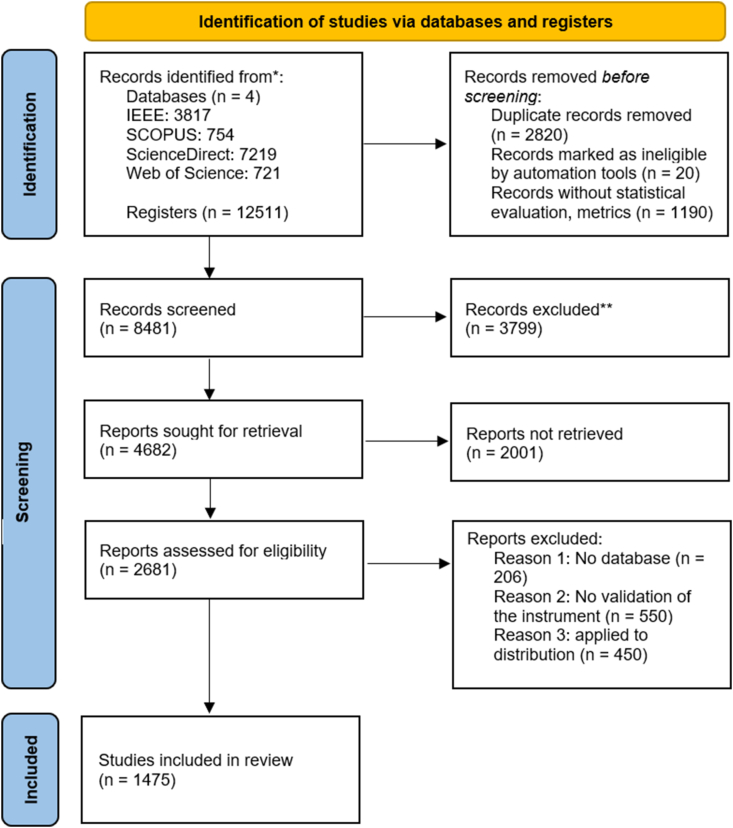
PRISMA-A methodology.

Fig. 1 identified 4 databases for the IEEE, Scopus, Elsevier, and Web of Science with 12511 registers, during the evaluation, the duplicate records removed 2820 articles, with 20 papers with ineligible by automation tool and 1190 articles without statistical evaluation and metrics.

Besides, in the screening stage were excluded 37900 articles for the evaluation, by applying these exclusion criteria, it remains focused, relevant, and of high quality, providing valuable insights into the evaluation of transformers in wind parks, and the excluded articles, as follows.

• Irrelevance to the Research Question: The articles didn't specifically focus on transformers in the context of wind parks. The papers discussed transformers in general but do not address their application in wind energy systems.
• Non-Primary Research: It is not considered the editorials, commentaries, opinion pieces, and non-peer-reviewed articles.
• Publication Date: It is not available before 1983, and papers published outside the defined date range for the review, especially if focusing on recent advancements and technology.

Technical Scope: About the papers didn't address the technical aspects of transformer performance, reliability, or maintenance in the context of wind parks.

### 2.2 Eligibility criteria

The eligibility criteria are established to guide the systematic review and meta-analysis focused on the accurate interpretation of Dissolved Gas Analysis (DGA) results in wind turbine transformers: This subsection included peer-reviewed journal articles, and database for 1475 articles included in the analysis. The publication date range is from January 1986 to the 2024. Only studies published in English are included, excluding those in other languages. There are no geographical restrictions, so studies conducted globally are included. The focus is on wind turbine transformers specifically addressing internal components and operational conditions, excluding studies on other types of transformers without specific reference to wind turbine applications. Studies applying DGA to wind turbine transformers, including those investigating internal auxiliary components, load break switches, and unique operational conditions, are included, while those applying DGA to transformers in general without specific mention of wind turbine transformers are excluded. Studies examining the impact of wind turbine operational conditions, such as load fluctuations and integration with power electronics, on DGA results are included, whereas those that do not consider the unique operational conditions of wind turbine transformers are excluded. The methodology criterion includes studies using quantitative and qualitative evaluation methods, including statistical analysis, trend evaluation, and risk bias assessment, excluding those lacking rigorous methodological approaches or sufficient data for analysis. Outcome measures include studies reporting on gas generation patterns, gas values, fault diagnosis accuracy, and the impact of internal auxiliary components on DGA results, excluding those without specific outcomes related to gas values or fault diagnosis in wind turbine transformers. The quality of data criterion includes well-documented methodologies, clear data sources, and comprehensive data analysis, excluding studies with poor documentation, unclear data sources, or inadequate analysis. Studies referencing established standards for mineral oil based on IEEE C57.104–2019 [5], ASTM D923 [7], and ASTM D3612 [8] are included, while those not aligning with recognized standards are excluded. Technological advancements include studies discussing new technologies, tools, or methods for improving DGA interpretation in wind turbine transformers, excluding those that do not contribute to technological advancements. By adhering to these eligibility criteria, the review aims to compile a comprehensive and high-quality set of studies that provide valuable insights into the interpretation of DGA results in wind turbine transformers, addressing the specific challenges posed by their unique operational and internal conditions.

And about not retrieved, is regarding as.

• Duplications: Duplicate publications of the same study, unless they provide additional, non-overlapping data.
• Outcomes and Metrics: Papers didn't consider for relevant outcomes or performance metrics pertinent to transformers in wind parks.
• Methodological Quality: Papers didn't meet predefined quality thresholds or criteria, such as sample size, validity of the data, or robustness of the analytical methods used.

### 2.3 Information source

The sources based on the analysis for IEEE (3817), Scopus (754), ScienceDirect – Elsevier (7219), and Web of Science (721), with a registered of 12511 articles.

### 2.4 Search strategy

**Fig. 2:**
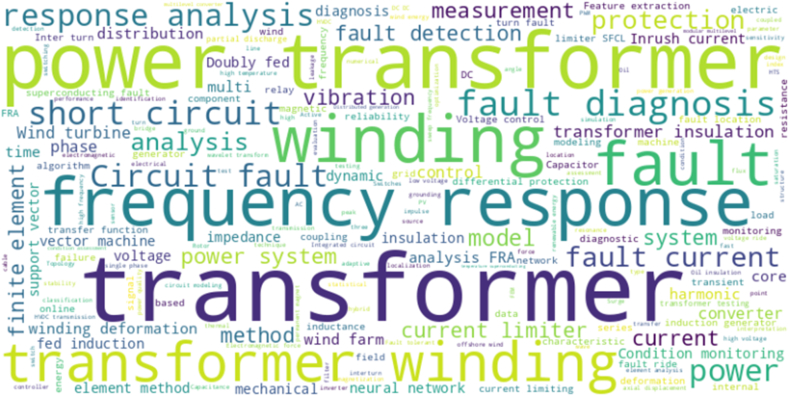
Words for the PRISMA-A methodology.

The analysis for 7080 words corresponding to the main keywords considered Fig. 2 and Table 1, in order to obtain the search string: (Transformer) AND (winding) AND (wind) AND (fault) OR ((diagnostic) OR (Current monitoring) OR (method) OR (algorithm)).

**Table 1:** Main keywords.

Perspective	Keywords
Population	Power transformers, winding, fault, wind farm, system
Intervention	Response analysis, measurement, diagnostic, fault diagnostic, core
Comparison	Feature extraction, short circuit, frequency response, condition monitoring, analysis FRA
Output	Method, neural network, vector machine.
Context	Winding deformation, fed induction, method, inductance, insulation, model

### 2.5 Selection process

**Fig. 3:**
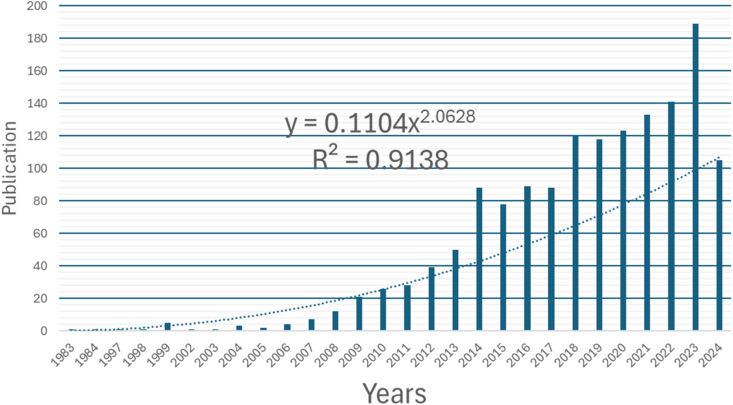
Results of the description.

The analysis for 7080 words corresponding to the main keywords considered Fig. 2 and Tables 1 and in order to obtain the search string: (Transformer) AND (winding) AND (wind) AND (fault) OR ((diagnostic) OR (Current monitoring) OR (method) OR (algorithm)). The results of the PRISMA-A process in Fig. 1, is 1475 research articles, with Eq. (1) based on the research articles per year from 1983 to 2024; the raise value is a constant 2.0628; therefore, it is growing faster than linear equation; in practical terms, the data set or a phenomenon that follows this power law, in Fig. 3 observed that doubling *X* increased *y* by a factor of 2.0628 ≈ 4.209.

(1) $$y=0.1104\;X^{2.0628}$$

The selection process considers the selection process for three scopes for the proceeding papers and international journal, in Table 2, as follows: The screening process with an automatic tool (One question), a full text screening process (three questions) and the inclusion criteria for the analysis (three questions).

**Table 2:** Selection process questions.

Scope	Question
Screening process with an automation tool	Are the results the process of the automatic tools with the main key words in Table 1 and phrases related to wind parks, transformer evaluation?
Full text screening process	What methodology is used for the evaluation?
	What is the evaluation of data quality?
	What is the relevance to the topics of wind park transformer evaluation and the specific issues under consideration?
Inclusion criteria	What are the papers that directly addressed the integration of renewable energy into the grid, particularly wind power?
	Are the papers that investigated issues related to the underutilization of wind farm transformers?
	Are the papers focused on Dissolved Gas Analysis and its interpretation in the context of wind turbine transformers?
Quality evaluation, for rigor	Is clear the robustness and appropriateness of the study design and methods?
	Is reliability and validity of the data included?
	Are the study's contribution to the field and its potential impact on transformer evaluation practices in wind parks?

### 2.6 Data collection process

The data collection process allows to evaluate transformers in wind parks, particularly in addressing underutilization, the application of DGA interpretation, a comprehensive data collection process and the integration of the database for all the research articles available, ensured the accuracy, relevance, and completeness of the data collected. The objective is collecting all the data from 1983 to 2024 that helps in evaluating the performance, reliability, and efficiency of transformers in wind parks, based on two databases, as follows.

• Historical Data: Past performance data, capacity factor records, and transformer utilization reports.
• Research Studies: Published research articles, conference papers, database available related to transformer evaluation and wind park performance.

The data collection process considers three steps with the data warehouse, cleaning, and normalization.

• Data Warehouse: To evaluate the centralized data warehouse to store all collected data, with csv files and the analysis by Python.
• Data Cleaning: Implement data cleaning protocols to ensure accuracy and consistency, removing any duplicates or errors, with a statistical analysis and outliers' evaluation.
• Data Normalization: Normalize data to ensure compatibility across different data sources and formats. The evaluation with the Yeo-Jhonson method.

### 2.7 Data items

The methodology is IEEE C57.104–2019 [5] for the DGA for mineral oil, it was retrieved and digitized historical performance and capacity factor data from archived records for seven elements, Hydrogen, Ethylene, Ethane, Carbon monoxide, Carbon dioxide, Methane, Acetylene, Oxygen and Nitrogen, for routine evaluation, and the Laboratory results for the available evaluation with the Duval methodology [5], IEC 60599 [10], Laboratec method [11], Rogers ratio [5], and CIGRE CS15 [12].

### 2.8 Risk bias

The integration of renewable energy, particularly wind power, into the grid presents significant challenges, particularly the high concentration of elements during normal operation and the new limits for fault detection. A critical issue is the underutilization of wind farm transformers, besides, the loading cycle. This proposal outlines a comprehensive approach for evaluating the risk of bias in studies investigating transformer performance in wind parks, particularly those exploring DGA. The objective is to evaluate the risk of bias in studies related to transformer evaluation in wind parks, to ensure the accuracy, reliability, and validity of collected data and analysis with a quality assessment to reporting of future studies to minimize bias.

The methodology used is the Cochrane Risk of Bias Tool [9], adapted for the context of engineering and technical studies, with the five process as follows, with the criteria in Table 3.

• Selection Bias: Assess whether the inclusion criteria for selecting studies and data sources were clearly defined and appropriate.
• Performance Bias: Evaluate the methods used to ensure consistent and accurate data collection.
• Detection Bias: Examine how outcome measures were defined and whether it was measured consistently across papers.
• Attrition Bias: Consider the completeness of data and how missing data were handled.
• Reporting Bias: Assess whether all relevant outcomes were reported and if there was selective reporting of results.

**Table 3:** Risk bias framework criteria.

Framework	Criteria
Selection bias	Were the criteria for including studies and data sources clearly defined and appropriate?
Performance bias	Were the methods for data collection consistent across different studies and wind parks?
Detection bias	Were the outcomes (DGA results) clearly defined and measured consistently?
Attrition bias	Were all expected data points reported, and were missing data adequately addressed?
Reporting bias	Were all relevant results reported transparently, without omitting critical findings?

The outcome the effect measure of the DGA is with 9 elements, in subsection 2.7, with the units’ parts per million (ppm), in Table 4.

**Table 4:** Current international standard for evaluation IEEE [5].

Elements	Unknown age	1–9 years	10–30 years	Higher 30
Hydrogen (H_2_)	80	75	75	100
Methane (CH_4_)	90	45	90	110
Ethane (C_2_H_6_)	90	30	90	150
Ethylene (C_2_H_4_)	50	20	50	90
Acetylene (C_2_H_2_)	1	1	1	1
Carbon monoxide (CO)	900	900	900	900
Carbon Dioxide (CO_2_)	9000	5000	10000	10000

According to Table 4, the risk bias for the unknown age for the transformers, and the diagnostic for values under this IEEE limits [5].

### 2.9 Synthesis method

**Fig. 4:**
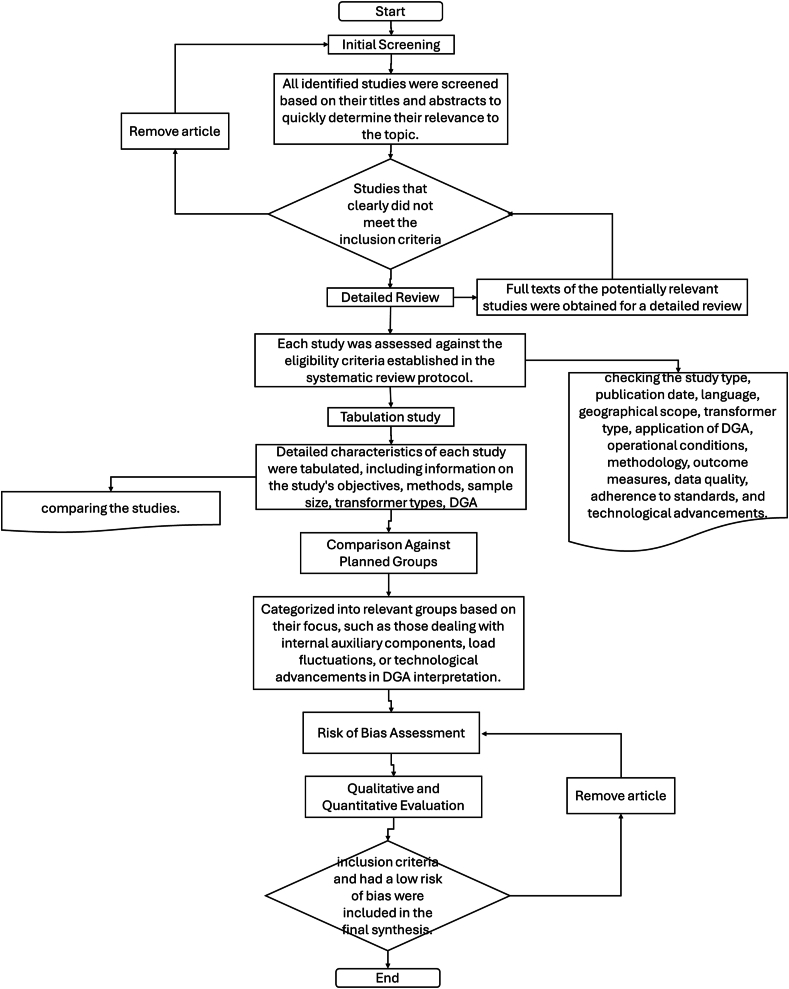
Processes used to decide which studies were eligible for each synthesis.

The review ensured that only high-quality, relevant studies were included in the synthesis, thereby providing robust and reliable insights into the interpretation of DGA results in wind turbine transformers. In Fig. 4, determined which studies were eligible for each synthesis in the systematic review and meta-analysis focused on the accurate interpretation of Dissolved Gas Analysis (DGA) results in wind turbine transformers, a multi-step process was employed. Initially, all identified studies were screened based on their titles and abstracts to quickly assess their relevance, excluding those that clearly did not meet the inclusion criteria, such as non-peer-reviewed articles and non-English publications. Subsequently, the full texts of potentially relevant studies were obtained for a detailed review, where each study was meticulously assessed against the eligibility criteria established in the systematic review protocol. This included evaluating the study type, publication date, language, geographical scope, transformer type, DGA application, operational conditions, methodology, outcome measures, data quality, adherence to standards, and technological advancements. Detailed characteristics of each study were tabulated, capturing key information such as objectives, methods, sample size, transformer types, DGA specifics, operational conditions, outcomes, and findings, which facilitated systematic organization and comparison. These tabulated characteristics were then compared against the planned groups for each synthesis, grouping studies based on specific aspects like DGA application type, operational conditions, and standards followed. A risk of bias assessment was conducted for each study using appropriate tools (Cochrane Risk), with studies showing high risk of bias flagged and carefully considered. Studies meeting all inclusion criteria and demonstrating low risk of bias were included in the final synthesis, where both qualitative and quantitative evaluations were performed. The qualitative synthesis provided a narrative summary of the findings, while the quantitative synthesis (meta-analysis) was conducted where data allowed for statistical pooling. To ensure reliability, decisions on study eligibility were made by at least two independent reviewers, with a third reviewer consulted in cases of discrepancy to reach a consensus. The process is as follows.

• Initial Screening:
  o All identified studies were screened based on their titles and abstracts to quickly determine their relevance to the topic.
  oStudies that clearly did not meet the inclusion criteria were excluded at this stage.
• Detailed Review:
  oFull texts of the potentially relevant studies were obtained for a detailed review.
  oEach study was assessed against the eligibility criteria established in the systematic review protocol. This involved checking the study type, publication date, language, geographical scope, transformer type, application of DGA, operational conditions, methodology, outcome measures, data quality, adherence to standards, and technological advancements.
• Tabulation of Study Characteristics:
  oDetailed characteristics of each study were tabulated, including information on the study's objectives, methods, sample size, transformer types, DGA application specifics, operational conditions, outcomes measured, and key findings.
  oThis tabulation helped in systematically organizing and comparing the studies.
• Comparison Against Planned Groups:
  oThe tabulated characteristics were compared against the planned groups for each synthesis. This involved grouping studies based on specific aspects such as the type of DGA application, the operational conditions of the wind turbine transformers, and the standards followed.
  oStudies were then categorized into relevant groups based on their focus, such as those dealing with internal auxiliary components, load fluctuations, or technological advancements in DGA interpretation.
• Risk of Bias Assessment:
  oA risk of bias assessment was conducted for each study using established tools suitable for the type of research with Cochrane Risk of Bias Tool modified for transformers.
  oStudies with a high risk of bias were flagged, and their impact on the overall synthesis was carefully considered.
• Qualitative and Quantitative Evaluation:
• Studies that met all inclusion criteria and had a low risk of bias were included in the final synthesis.
• Both qualitative and quantitative evaluations were performed, with qualitative synthesis providing a narrative summary of the findings and quantitative synthesis (meta-analysis) conducted where data allowed for statistical pooling.
• Consensus report:
  oDecisions on study eligibility were made by at least two independent reviewers to minimize bias.
  oIn cases of discrepancy, a third reviewer was consulted, and discussions were held until consensus was reached.

### 2.10 Certainty assessment

The reporting method considers the evaluation of the database, however, the unbalance database The Yeo-Johnson transformation is defined differently for positive and non-positive values of the data [13]. The transformation function T(y, λ) for a given data point *y* and a transformation parameter *λ* is in Eq. (2); with y ≥ 0.

(2) $$T\left(y,\lambda \right)=\left\{ \begin{array}{c} \left({\left(y+1\right)}^{\lambda }‐1\right)/\lambda ,\text{with}\;\lambda \neq 0\\ \log\left(y+1\right),\text{with}\;\lambda =0 \end{array}\right.$$

And the for y < 0, Eq. (3).

(3) $$T\left(y,\lambda \right)=\left\{ \begin{array}{c} \left({‐\left(\left|y\right|+1\right)}^{2‐\lambda }‐1\right)/\left(2‐\lambda \right),\text{with}\;\lambda \neq 2\\ ‐\log\left(\left|y\right|+1\right),\text{with}\;\lambda =2 \end{array}\right.$$

With the results, the gaussian distribution symmetric about the mean. It is characterized by its mean (*μ*) and standard deviation (*σ*). The probability density function (PDF) of a Gaussian distribution based on Eq. (4). The goal of the Yeo-Johnson transformation is to make the transformed data *T*(*y*;*λ*) approximate a normal distribution, according to Reference [13].

(4) $$f\left(x,\mu ,\sigma^{2}\right)=\frac{1}{\sqrt{2\pi \sigma^{2}}}\exp\;\left(‐\frac{{\left(x‐\mu \right)}^{2}}{2\sigma^{2}}\right)$$

## 3 Results

During the last years, several initiatives increased the power generation as the yaw performance he model's versatility extends to optimizing yaw angle configurations using genetic algorithms, achieving prediction accuracy levels comparable to industrial standard tools with a relative accuracy of over 99 % [15]. In rigorous tests, the transformer surrogate model maintained high accuracy across successive generations of genetic algorithms. For example, when comparing the top-performing yaw configurations, the relative errors were 99.7 %, 98.1 %, and 96.3 % for the cluster, multi-string, and Horns Rev layouts, respectively. The model significantly reduced computation time, completing genetic algorithm runs in approximately 1 min for wind farms with 45, 55, and 80 turbines, compared to 25.5, 30.4, and 44.3 min using traditional methods.

In 2024, the focus was centered in the dynamic thermal rating (DTR) for transformers connected to wind farms by accurately predicting load profiles considering wake effects and turbine availability. It emphasizes that dynamic thermal rating allows transformers to operate beyond their nameplate rating under real-time weather and load conditions. The methodology involves a correction method for wake loss computation and accounts for the intermittent nature of wind affecting turbine availability. In Reference [14] has the key findings from a case study indicate that both wake effects and fluctuating turbine availability reduce the duration transformers operate at full load, thus lowering their aging rate. Wake effects are notably influential when wind speeds are in specific ranges, impacting wind power generation and consequently the load on wind farm export transformers (WFETs). The improved prediction models, validated against measured data, show that incorporating these factors allows for more accurate wind power and load profile estimations. Therefore, the expansion of wind farm capacity by 74%–77 % without exceeding the transformer aging rate seen at rated conditions. This is a significant improvement over the initial 63 % expansion capacity observed without these considerations. The study also demonstrates that high wind speeds, which lead to reduced turbine availability, mitigate the risk of overloading transformers by lowering the load profiles. In this case, the contribution was that integrating turbine availability and wake effects into load profile estimations can significantly improve the operational efficiency and longevity of WFETs. This approach suggests that it is possible to downsize transformers during the planning stage, thereby optimizing costs while maintaining acceptable aging losses. The methodology is versatile and, with appropriate adjustments, can be applied to offshore wind farm substations to optimize transformer ratings and reduce construction complexities [14].

In this case, the DTR increased in the transformers, in innovative method to accurately assess the operating status of power transformers, crucial components in the power grid. With the rise of renewable energy, transformers face challenges like overload, harmonics, and short circuits. The proposed method employs an optimal cloud entropy parameter calculation and a variable weighting approach to refine the grey-cloud evidence model. Through multi-source information fusion, the method synthesizes results from various tests to provide state awareness of transformers. In comparative analysis, the method demonstrated superior performance. When compared to the grey clustering method and the variable weight grey cloud model, the proposed method achieved evaluation results that closely matched the actual operating conditions of transformers. For instance, the grey clustering method correctly evaluated 8 out of 10 transformers, however, misjudged the conditions of the first and second transformers. Similarly, the variable weight grey cloud model inaccurately assessed the sixth and tenth transformers. In contrast, the proposed method consistently produced results that aligned with the true state of all 10 transformers evaluated, described in Table 5.

**Table 5:** Oil-immersed problems in wind turbine transformers [16].

Metric	Description
Comparison Methods	Proposed method vs. variable weight grey cloud model
State Levels Evaluated	Good, General, Exceptions, Severity
Transformers Evaluated	10
Accurate Evaluations (Grey Clustering)	8 out of 10 transformers
Discrepancies (Grey Clustering)	Transformer 1 (Good), Transformer 2 (Abnormal)
Accurate Evaluations (Grey Cloud Model)	8 out of 10 transformers
Discrepancies (Grey Cloud Model)	Transformer 6, Transformer 10
Evaluation Results Consistency	Proposed method aligns with actual state for all 10 transformers
Experiment 1	Consistency with actual transformer conditions through 3 sets of experiments
Experiment 2	Consistency with actual conditions using data from 10 transformers
Impact Considerations	Wind power generation, high penetration of new energy sources
Evaluation Index	Combines experimental data, monitoring data, and external environmental data
Key Features of Proposed Method	- Dynamic weight allocation - Multi-source information fusion - Avoids high-conflict evidence fusion - Enhances accuracy of transformer status assessment

### 3.1 Study selection

The PRISMA-A process started with four databases: IEEE (3817), Scopus (754), ScienceDirect (7219) and Web of Science (721) with 12511 registers with a range from 1986 to 2024, and its outputs were 1475 registered included in this review article; with the 7080 words corresponding to the main keywords for the search strategy. With this research articles, therefore, the database obtained 4810 DGA samples for wind park, for faults and normal conditions associated to historical data and research studies by using the processes used to decide which studies were eligible for each synthesis in Fig. 4. The exclusion considered oil applied to solar, hydro, geothermal technologies.

All the gas evaluation, for instance the Hydrogen (H_2_) gas in wind turbine transformer oil is detected through Dissolved Gas Analysis (DGA), performed according to IEC 60567 [56], as outlined in section 9.7 of IEC 62975:2021 [57]. This analysis involves extracting the insulating liquid and analyzing it for the presence of gases, including hydrogen, which is a key indicator of potential faults like partial discharges or overheating within the transformer. The DGA process is part of a broader set of physicochemical tests recommended for the insulating liquid, including assessments of dielectric strength, moisture content, acidity or neutralization number, interfacial tension, dissipation factor (Tan Delta), and oxidation inhibitor content, as specified by various IEC standards.

### 3.2 Evolution of the state of art

In Table 6, the Probabilistic Machine Learning Model, utilizing Generative Adversarial Networks (GAN), achieves an error rate of 0.47 % for median value predictions and maintains 80 % prediction interval errors within 6%–7%. This model offers accurate lifetime predictions for transformers while significantly reducing simulation time and memory requirements. However, it necessitates extensive data and further testing for end-to-end operation, with additional research needed to fully implement GANs effectively.

**Table 6:** Evolution of art.

Method	Metrics	Contribution	Limitation
HTS Transformer Design, validated with Thermal and Electromagnetic Analysis [18] (2024)	250 MVA, 33/220 kV; Efficiency: 99.7 %; Weight: 98,000 kg; HSP temp: 71.34 K (normal), 127.2 K (fault). AC loss, temperature distribution of windings	Reduced weight by 71 % compared to conventional transformers; Improved efficiency by 0.46 %; Enhanced cooling with porous coating; Reduced AC loss by 50 % and HSP temp by 29 %	12 % heavier than a 100 MVA HTS transformer due to FD and cooling system; Specific operating conditions need consideration. Limitation: Complexity in heat transfer analysis during fault conditions; Need for accurate micro-meshing to analyze porosity effects on heat transfer
Probabilistic Machine Learning Model [17] Generative Adversarial Network (GAN) (2023)	Error of 0.47 % for median value; 80 % prediction interval errors within 6%–7%. Potential for exhaustive exploration and testing	Provides accurate lifetime predictions for transformers with significantly reduced simulation time and memory requirements	Requires extensive data and further testing to ensure end-to-end operation. GAN requires further research and development to be effectively implemented
Grey-box model for transformer temperature prediction [19] (2023)	MAE: 0.39–0.63 °C, Max Error: 1.69–2.47 °C, Prediction Horizon: 6h	High accuracy in temperature prediction, non-intrusive application, real-time forecasting	Requires re-optimization of parameters, needs an algorithm for online implementation
Thermal-Hydraulic Model (TH), Experimental Validation with Transformer Prototype [24] (2023)	Maximum relative error: 1.31 %; with 29 optical fiber sensors	Accurate prediction of oil flow and temperature distribution in power transformers. Validates the model's accuracy with detailed measurements across multiple points	Model approximations may not capture singular and local phenomena; some discrepancies due to practical tolerances. Slight deviation from model results; some sensors damaged during manufacturing
Nonlinear autoregressive neural networks [23]	RMSE: 1.6 °C	Accurate thermal modeling of high rating transformers	Higher errors compared to the proposed model, requires large data set, suitable for monitoring
Fuzzy tree method [20] (2021)	RMSE: 0.7 °C	Accurate prediction of winding hot-spot temperature	Less accurate compared to the proposed model, limited to monitoring, requires internal measurements
New thermal method [21] (2021)	Squared Average Error: 1.26–1.36 °C, Max Error: 4.42–5.54 °C	Improved accuracy over IEEE C57.91 Standard methods	Higher errors compared to the proposed model, requires internal measurements, limited to monitoring
Hot-spot prediction model [20] (2021)	RMSE: 0.03–0.08 °C	Very high accuracy in hot-spot temperature prediction	Specific to dry-type transformers, not suitable for step-ahead predictions, requires internal measurements
Artificial neural network [22] (2020)	Mean Squared Error: 2.71 %	Accurate temperature distribution prediction for dry-type transformers	Requires large data set and 3D data, suitable for transformer design purposes

In contrast, the HTS Transformer Design validated with Thermal and Electromagnetic Analysis boasts a 250 MVA capacity and 99.7 % efficiency, while reducing weight by 71 % compared to conventional transformers. Despite these advancements, it is 12 % heavier than a 100 MVA HTS transformer due to the FD and cooling system, and it requires precise micro-meshing for accurate porosity effect analysis during fault conditions. The Grey-box model for transformer temperature prediction demonstrates high accuracy with a mean absolute error (MAE) of 0.39–0.63 °C and a maximum error of 1.69–2.47 °C, over a 6-h prediction horizon, making it suitable for real-time forecasting without intrusive application. Nevertheless, it requires parameter re-optimization and an algorithm for online implementation. The Fuzzy tree method predicts winding hot-spot temperatures with a root mean squared error (RMSE) of 0.7 °C but is less accurate compared to the proposed model and limited to monitoring with internal measurements.

In [39] provides an overview of three key methods for fault diagnosis in oil-filled electrical equipment: the IEEE Key Gas Method, IEC 60599 Three Gas Ratio Method, and the Graphical Method known as the Duval Triangle. A traditional method is IEEE Key Gas Method has correlates seven gases (H_2_, CH_4_, C_2_H_6_, C_2_H_4_, C_2_H_2_, CO, CO_2_) with specific fault types (overheating of oil, overheating of cellulose, partial discharge, and arcing), therefore, it relies on qualitative relationships rather than numerical correlations, which has led to significant inaccuracies (up to 58 % incorrect diagnoses) according to studies. Besides, IEC 60599 with Three Gas Ratio Method, classified faults into five major categories (partial discharges, low energy discharges, high energy discharges, and thermal faults at various temperatures). It employs numerical thresholds to match gas ratios against fault patterns, yet it can still result in up to 8 % incorrect diagnoses and 15 % unresolved cases.

On the other hand, graphical Method as Duval Triangle, it used the CH_4_, C_2_H_2_, and C_2_H_4_ gases. It provides a visual map where different fault types occupy distinct regions. This method boasts high accuracy (up to 96 %) but may suffer from overlapping fault indications and lacks a direct visualization of fault evolution over time. In 2016, the new technique used the normalizes CH_4_, C_2_H_2_, and C_2_H_4_ gases and applies fuzzy trapezoidal membership functions derived from Duval Triangle mappings, besides, in 2017, the research article in Ref. [12].

For instance, the Duval Triangle method (90.6 % accuracy in tested cases); and IEC 60599 method correctly identifies 77.78 % of faults, while the new technique achieves 90.6 % accuracy, matching the Duval Triangle's performance.

In [40], triangles and pentagons in fault diagnosis, as outlined in Refs. [41,42], provides a structured approach depending on the complexity and nature of faults identified in oil-filled electrical equipment. For single fault identification, placing DGA points in Triangle 1 or Pentagon 1 suffices to classify one of the six basic fault types. However, for more detailed analysis involving additional subtypes of thermal faults, Pentagons 2, along with Triangles 4 or 5, are recommended. When multiple faults are suspected, comparing diagnoses from both Pentagons and Triangles becomes crucial; discrepancies often indicate the presence of multiple simultaneous faults.

The New thermal method improves accuracy over IEEE C57.91 Standard methods with squared average errors of 1.26–1.36 °C and maximum errors of 4.42–5.54 °C, though it still exhibits higher errors than the proposed model and relies on internal measurements. The Hot-spot prediction model offers exceptional accuracy with an RMSE of 0.03–0.08 °C, but it is specifically tailored for dry-type transformers and not suitable for step-ahead predictions. The Artificial neural network achieves a mean squared error of 2.71 %, providing precise temperature distribution predictions for dry-type transformers, but requires large datasets and 3D data, making it more applicable for transformer design purposes. Lastly, Nonlinear autoregressive neural networks exhibit an RMSE of 1.6 °C, ensuring accurate thermal modeling for high rating transformers, though they demand large datasets and are primarily used for monitoring.

The last evaluation during 2019, doble introduced a daily gasification for the evaluation of failure modes in mineral oil.

• Arc: It should be monitored.
• Thermal fault (T1 to T3) and partial discharge fault (DP).
  o More than 100 ppm/day, it is a signal of sudden fault.
  o Between 3 ppm/day to 99.9 ppm/day. It is a risk condition.
  o Between 1 and 2.99 ppm/day; it is a monitoring condition.
  o Normal condition for lower values than 1 ppm/day.

Besides, usually, during thermal fault or partial discharge, the first option is the oil treatment in order to treat the gas element as Hydrogen and Ethylene and Methane for thermal faults, or hydrogen and methane for arc or partial discharge; the gas accumulated in the kraft didn't disappear and the degasification process didn't eliminate all the combustible gas, even worse, the impregnated oil in the kraft included Methane, Ethylene and Hydrogen, in a rebound effect in the oil. various failure theories in transformer insulation, focusing on the quantitative effects of adding corn oil to mineral oil. Four main failure theories are discussed: Electronic Failure Theory, Gas Failure Theory, Solid Particle Failure Theory, and Liquid Ball Failure Theory.

Experimental methods involved testing different compositions of corn oil mixed with mineral oil to evaluate dielectric strength. Results indicate that adding 20 % corn oil to mineral oil (sample MJ20) optimally increases breakdown voltage to 30.66 kV/2.5 mm, meeting standard specifications. However, higher corn oil compositions decrease breakdown voltage, indicating reduced insulation strength. Chemical-physical properties such as viscosity and density also increase with higher corn oil content. The study suggests 20 % corn oil as a viable alternative for transformer oil applications at 2.4 kV working voltage, aligning with NESC standards [25].

### 3.3 Results discovered in the database for wind park transformers

**Fig. 5:**
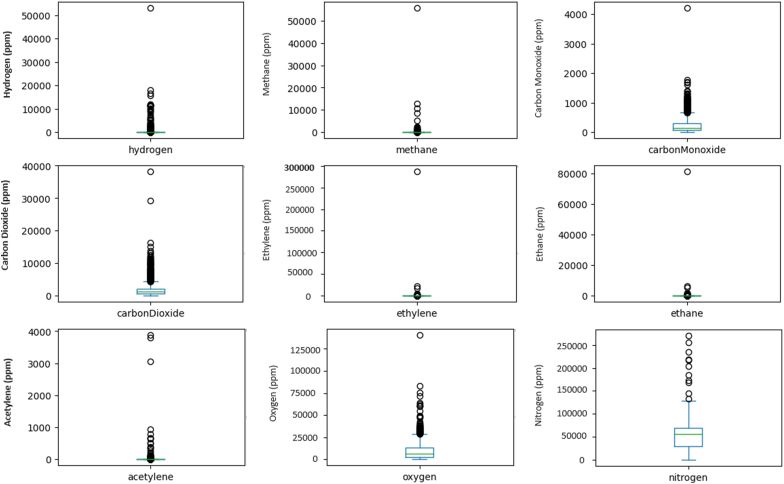
Boxplot for the analysis, regarding the faults and normal condition.

The database obtained 4810 samples of DGA for wind parks, for faults and normal condition from the 1475 research articles in the PRISMA-A method; therefore, Table 5, statistical data for various elements found in a set of samples, presenting several descriptive statistics for each element, an unbalance dataset: hydrogen, methane, carbon monoxide, carbon dioxide, ethylene, ethane, acetylene, oxygen, and nitrogen. For hydrogen, with 3686 samples, the mean concentration is 158.23 ppm, a standard deviation of 1243.21 ppm, and values ranging from 0 to 52940 ppm. The 25th, 50th, and 75th percentiles are 6 ppm, 15 ppm, and 36 ppm, respectively. Methane, with 3953 samples, has a mean of 49.68 ppm, a standard deviation of 941.23 ppm, and a range from 0 to 55617 ppm. The percentiles are 3 ppm, 6 ppm, and 12 ppm. Carbon monoxide, from 3972 samples, shows a mean of 237.88 ppm, standard deviation of 255.63 ppm, and values from 0 to 4199 ppm, with percentiles at 69 ppm, 148 ppm, and 307 ppm. Carbon dioxide, with 3973 samples, has a mean of 1663.06 ppm, a standard deviation of 1799.69 ppm, and ranges from 0 to 38115 ppm, with percentiles at 608 ppm, 1175 ppm, and 2147 ppm. Ethylene, with 3549 samples, exhibits a mean of 108.13 ppm, a high standard deviation of 4831.25 ppm, and ranges from 0 to 286384 ppm, with percentiles at 0 ppm, 1 ppm, and 4 ppm. Ethane, from 3661 samples, shows a mean of 56.41 ppm, standard deviation of 1349.79 ppm, and a range from 0 to 81123 ppm, with percentiles at 1 ppm, 2 ppm, and 12 ppm. Acetylene, with 2581 samples, has a mean of 8.09 ppm, a standard deviation of 128.52 ppm, and values ranging from 0 to 3871 ppm, with percentiles at 0 ppm, 0 ppm, and 0 ppm. Oxygen, from 3960 samples, shows a mean of 8690.35 ppm, standard deviation of 8713.26 ppm, and ranges from 0 to 140321 ppm, with percentiles at 2168 ppm, 6224.5 ppm, and 12828.25 ppm. Nitrogen, with 3960 samples, exhibits a mean of 50921.34 ppm, a standard deviation of 25599.89 ppm, and values from 0 to 269548 ppm, with percentiles at 29403.75 ppm, 56256.5 ppm, and 69825.75 ppm; in Fig. 5.

**Fig. 6:**
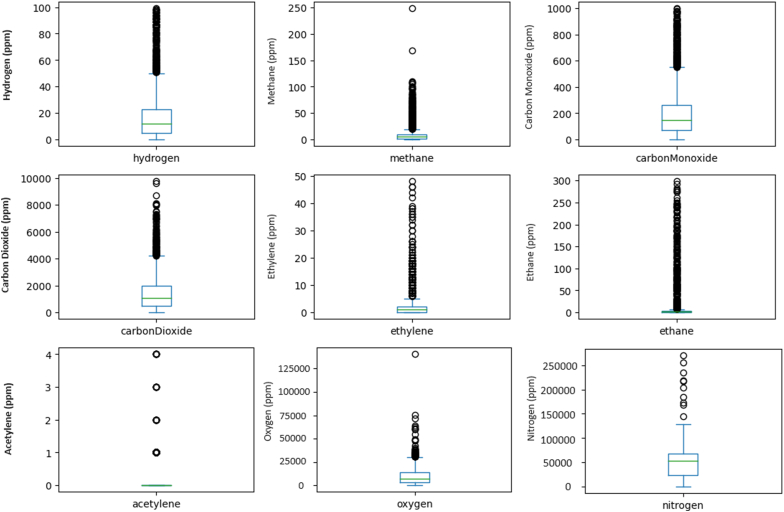
Boxplot for the analysis, for limits in the normal condition.

Besides, in Fig. 6 has the following description.

• For hydrogen, with 1920 samples, the mean concentration is 15 ppm, and values ranging from 0 to 100 ppm. The 25th, 50th, and 75th percentiles are 10 ppm, 16 ppm, and 22 ppm, respectively.
• Methane, with 1500 samples, has a mean of 45 ppm, and a range from 0 to 250 ppm. The percentiles are 6 ppm, 11 ppm, and 24 ppm.
• Carbon monoxide, from 1188 samples, shows a mean of 190 ppm, and values from 0 to 1000 ppm, with percentiles at 80 ppm, 195 ppm, and 326 ppm.
• Carbon dioxide, with 2990 samples, has a mean of 1800 ppm, and ranges from 0 to 9900 ppm, with percentiles at 700 ppm, 1240 ppm, and 2250 ppm.
• Ethylene, with 991 samples, exhibits a mean of 11 ppm, and ranges from 0 to 50 ppm, with percentiles at 1 ppm, 8 ppm, and 25 ppm.
• Ethane, from 991 samples, shows a mean of 35 ppm, and a range from 0 to 300 ppm, with percentiles at 10 ppm, 20 ppm, and 37 ppm.
• Acetylene, with 1409 samples, has a mean of 0.5 ppm, and values ranging from 0 to 4 ppm, with percentiles at 0 ppm, 0 ppm, and 0 ppm.

**Fig. 7:**
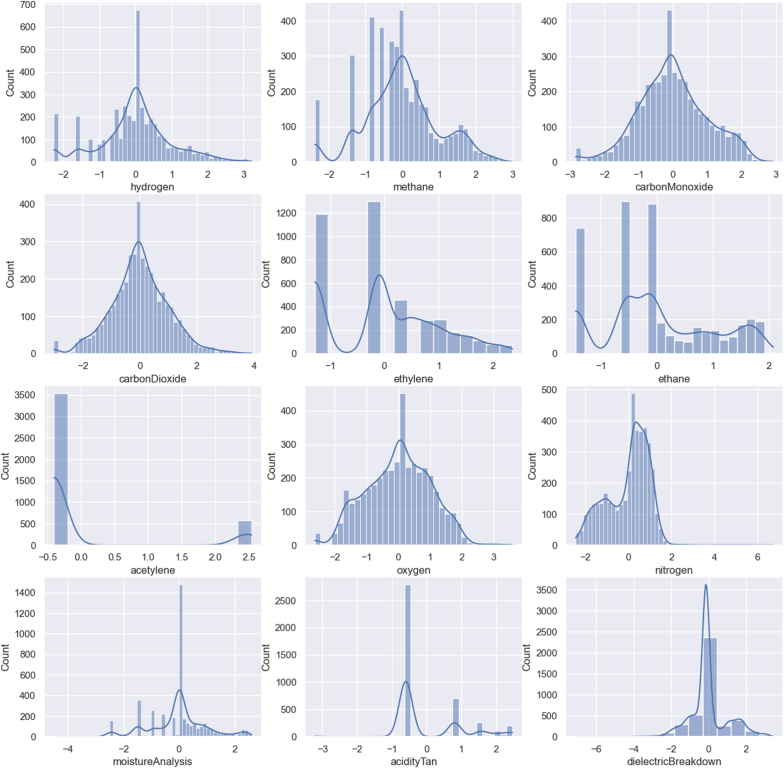
Gaussian distribution with Yeo-Johnson transformation results.

The Yeo-Johnson transformation is a statistical technique used to transform data into a Gaussian (normal) distribution. It is particularly useful when dealing with data that do not follow a normal distribution. The transformation can be applied to both positive and negative values, which distinguishes it from the Box-Cox transformation that only works with positive values, in Fig. 7.

**Fig. 8:**
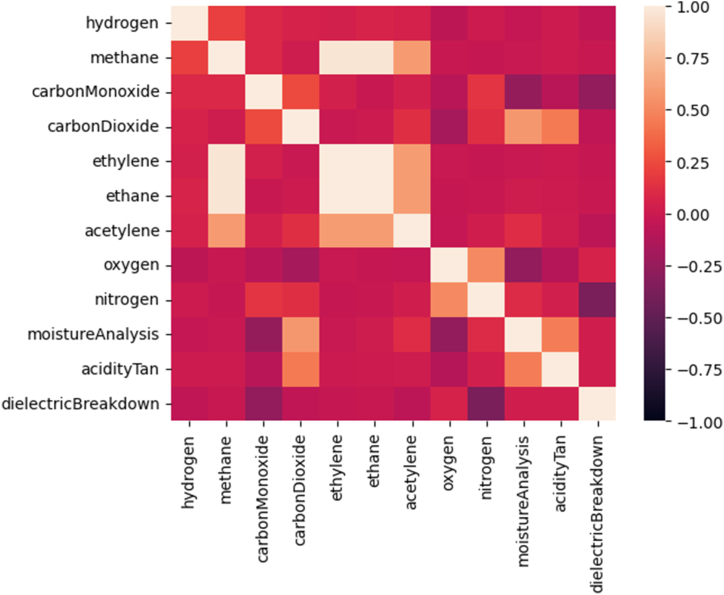
Correlation matrix for dissolved gas analysis and physical/chemical test.

In the evaluation of the correlation has the following behavior according to Fig. 8.

• Methane with high correlation of 98.5 % with Ethylene and Ethane, based on partial discharge with high temperature.
• Ethylene and Ethane with correlation of 99.1 %, based on thermal faults.
• Acethylene with correlation of 90.2 % and Ethylene, besides 91.8 % with Ethane, based on arc faults or partial discharge.
• Carbon monoxide with high correlation of −75.9 % with dielectric breakdown, regarding the kraft properties, based on thermal fault in the Kraft.
• Carbon dioxide with high correlation of 92.9 % with moisture, based on kraft degradation.

In Reference [26], the transition from petroleum-based mineral oil to biodegradable alternatives in power system applications, focusing on palm fatty acid ester (PFAE). The research investigates the partial discharge (PD) characteristics of PFAE compared to soybean-based oil and mineral oil, under varying electrical stresses and ageing conditions. Experimental findings reveal that PFAE exhibits PD magnitudes slightly lower than mineral oil, indicating its potential as a viable transformer oil. The study underscores the importance of biodegradable oils in mitigating environmental risks associated with traditional mineral oils. The experimental setup adheres to IEC 60156 standards, ensuring test cell cleanliness and electrode gap precision (2.5 mm ± 0.05 mm). Test cells are preconditioned to minimize moisture content and maintain consistent dielectric properties during testing, in this sense, oil samples undergo rigorous preconditioning to stabilize humidity levels and reduce contaminants using magnetic stirrers at 50 °C. Therefore, the analysis reveals consistent PD behaviors across oil samples, with PFAE and mineral oil exhibiting similar PD charge trends at both voltage levels. Soybean-based oil shows higher PD activity compared to PFAE and mineral oil, suggesting superior performance of PFAE in PD suppression.

#### 3.3.1 Risk bias in the trends

On the other hand, the trends investigated the use of palm oil, coconut oil, and virgin coconut oil as alternatives to petroleum-based transformer oils. The results demonstrate that virgin coconut oil exhibits the highest breakdown voltage, lowest moisture content, and lowest viscosity among the tested oils, indicating superior performance as an insulating liquid. These findings highlight virgin coconut oil's potential as an environmentally friendly and renewable option, superior to palm and coconut oils. However, the paper acknowledges that the high cost of these vegetable oils, due to their use in the food industry, and their tendency to absorb moisture and oxidize, pose challenges for large-scale application in power systems, therefore, the contribution of the [27] lies in presenting virgin coconut oil as a promising biodegradable and renewable alternative to traditional mineral oils in transformers. The qualitative analysis shows clear performance advantages in dielectric strength, moisture content, and viscosity. However, the evaluation might exhibit a risk bias, as it strongly favors virgin coconut oil based on these parameters without equally weighing the economic and practical challenges. A balanced assessment should consider the cost, long-term stability, and scalability of using vegetable oils in transformers.

During 2024, the use of canola oil as a sustainable alternative to traditional petroleum-based insulating oils for high-voltage transformers was investigated in Ref. [28]; its highlights are the high-temperature stability, fire safety, and excellent cold flow properties. However, it also notes practical limitations, including its susceptibility to oxidation and challenges in low-temperature flow. In Ref. [28] incorporated additives to improve oxidation stability and using winterization techniques to lower the pour point, however, it concluded that while canola oil shows significant promise as an insulating liquid, ongoing research is needed to address its limitations and ensure its viability in large-scale applications. The contribution of this study lies in its comprehensive analysis of canola oil's potential as a transformer insulating liquid, focusing on its dielectric properties, fire safety, and environmental benefits. Qualitatively, the study provides valuable insights into the advantages and challenges of using canola oil, suggesting practical solutions for its limitations. However, the evaluation may present a risk of bias, as it emphasizes the positive attributes of canola oil without equally weighing the economic and practical challenges, such as the cost of modifications for retrofitting existing systems and the complexities of oxidation stability. A balanced assessment should consider these factors to provide a holistic view of canola oil's feasibility as a sustainable insulating liquid. The transformers that have been developed for these applications do not have an expansion tank in their original designs, and seek to reduce the standard dimensions with an integrally filled corrugated tank design, however the information found shows an increase in hydrogen of more than 1000 ppm, ethane of more than 500 ppm, and in some cases of sudden failure with acetylene greater than 3 ppm and activation of the Buchholz protection, with common failure modes of electric arcs and partial discharge.

Furthermore, in 2024 a novel probabilistic feature-selection approach to improve the lifetime estimation of power transformers operated in renewable power plants, however, it addresses the challenges posed by the increased penetration of renewable energy sources, which introduce weather dependency and dynamic operational phenomena affecting asset lifespan. By weighting and selecting features probabilistically through a heuristic and iterative process, the proposed approach enhances the accuracy and robustness of health monitoring. In Ref. [29] evaluated in two countries in Spain and Australia. The contribution is substantial as it provides a novel methodology for improving asset lifetime estimation, crucial for the reliability and efficiency of RES operations. The qualitative information includes the adaptation to dynamic environments, consistent reduction of prediction errors, and relevance in feature selection, enhancing the overall asset management process. Regarding bias, the abstract's reliance on results from specific photovoltaic power plants may introduce a risk of bias, as the generalizability to other RES components or environments isn't empirically verified. However, the claim of generalizability is supported by a logical extension of the approach rather than empirical evidence across diverse scenarios.

As a results of the nanofluids used for insulating oils in transformers, several methodologies offer distinct benefits and limitations. One prominent method involves the addition of surfactants, which act as dispersants to prevent nanoparticle aggregation within the nanofluid. In Ref. [38], the use of surfactants like benzalkonium chloride (BAC) and benzethonium chloride (BZC), has demonstrated significant improvements in dispersion stability, as seen in studies with silicon dioxide nanoparticles in Terminal 66 oil. However, surfactants can be susceptible to thermal degradation at elevated temperatures, potentially leading to foaming issues and alterations in thermophysical properties such as increased viscosity and reduced thermal conductivity. Another approach, surface modification through functionalization of nanoparticles, offers a surfactant-free alternative for achieving long-term stability. By attaching functional groups to nanoparticles, this method promotes self-stabilization, mitigating the drawbacks associated with surfactant use. Techniques like ultrasonic agitation further enhance stability by disrupting attractive forces among nanoparticles, although optimal sonication parameters require careful calibration to avoid unintended particle size variations. Adjusting pH levels in nanofluids represents another effective strategy, leveraging electrokinetic properties to control nanoparticle dispersion. Studies have shown that nanofluids with optimized pH levels exhibit enhanced stability, crucial for maintaining long-term performance in transformer applications. Each method presents distinct quantitative metrics for stability enhancement, ranging from dispersion efficiency and thermal stability to particle size distribution and electrokinetic behavior, underscoring their diverse applications in advancing nanofluid technology for transformer insulation.

#### 3.3.2 Trends of the methods for predictive maintenance regarding oil evaluation

In [30] provides an in-depth examination of the advancements in mechanical state recognition of oil-immersed transformers using vibration signals. This research is presented from a novel sensor-oriented perspective, addressing sensor deployment, specialization, and equipment integration, which are pivotal in enhancing the accuracy and reliability of mechanical state diagnostics. The paper discusses various signal processing and feature selection techniques, comparing them with those used in rotating machinery. Additionally, it introduces emerging technologies such as Operational Modal Analysis and multisource data fusion, which offer promising solutions to existing challenges in transformer diagnostics. By integrating multiple data sources and employing advanced sensors, the research aims to improve predictive maintenance strategies, reduce maintenance costs, and enhance transformer reliability, ultimately contributing to the global efforts in mitigating greenhouse emissions. The qualitative information highlights the comprehensive nature of the study, the novel approach taken, and the introduction of emerging technologies that address current limitations. However, the risk bias based on the optimistic portrayal of emerging technologies like Modal Analysis and multisource data fusion without equally discussing their limitations or potential drawbacks. While the paper acknowledges challenges such as limited rule transferability and weaknesses in vibration characteristics, a more critical evaluation of the feasibility and practicality of these emerging solutions in real-world applications could provide a more balanced perspective. This ensures that readers are aware of both the benefits and the potential limitations of the proposed advancements. The methodologies applied in the image analysis of vibration signals from transformers and rotating machinery showcase a range of advanced techniques and their respective performances. Time-frequency diagrams utilizing Convolutional Neural Networks (CNN) achieve a high accuracy of 92.74 % [31,32], while Double Branch CNN demonstrates even greater accuracy at 98.3 % [33,34]. The Markov Transition Field, implemented with a Multi-parallel CNN approach, achieves notable performance of 96.15 % [35,36]. Hybrid image approaches, such as using Gramian Angular Summation Field in Semisupervised Learning, achieve exceptional accuracy of 99.56 % with partial labeling [37]. These results underscore the effectiveness of AI-based methods in enhancing the analysis of vibration signals, highlighting their potential for improving diagnostic capabilities in mechanical systems.

In Table 7, methods for interpreting dissolved gas analysis (DGA) in transformers and electrical equipment. The Duval Pentagon 2 and Triangle 5 (2023) method provides a structured approach for identifying faults with carbonization of paper, utilizing DGA points plotted in Pentagon 2 and Triangle 5 to categorize fault types based on detection above 90 % typical concentration values for faults and below for stresses, relying on visual inspection and available algorithms for DGA plotting [43]. Pentagons 1 and 2 (2022) offer graphical methods for visual identification of gas formation patterns in transformers, distinguishing between thermal and electrical faults, with similar concentration thresholds as the Duval Pentagon 2 method. The Key Gas Method (KGM) (2022) interprets fault gases based on individual gas presence, focusing on key gases like C_2_H_2_ to indicate fault severity, using specific threshold values for fault type determination. The Duval Pentagon Approach with CNN (2022) integrates all five hydrocarbon gases for fault identification, including H_2_ and C_2_H_6_, achieving high validation accuracy with a machine learning model, yet potentially biased towards published fault databases and specific fault scenarios [[44], [45], [46]]. Lastly, Duval Pentagon 2 (2021) identifies gassing patterns in transformers, quantifying gas levels and relying on laboratory tests and visual inspections for pattern recognition, essential for distinguishing fault types and ensuring operational reliability [47].

**Table 7:** Trends in Duval analysis and its limits.

Method	Contribution	Metrics	Limits	Bias
Hybrid Approach (2024) [50]	Proposed to identify a wider range of oil-immersed transformer faults.	Accuracy of identifying all fault types compared to IEC 60599, gas ratio, and limit-based methods.	Achieves 100 % accuracy in fault identification tasks.	Improves accuracy by 14.46 % compared to existing methods.
Using Duval Pentagon 2 and Triangle 5 (2023) [43]	Provides a structured approach for identifying faults C with carbonization of paper	Uses DGA points plotted in pentagon 2 and triangle 5 to categorize fault types.	Detection above 90 % typical concentration values (faults), below 90 % (stresses).	Relies on visual inspection for fault validation; algorithms available for DGA plotting.
Using Pentagons 1 and 2 (2022) [44]	Graphical methods for interpreting DGA in transformers and electrical equipment.	Visual identification of gas formation patterns (thermal and electrical).	Above 90 % typical concentration values for faults; below for stresses.	Relies on visual validation and post-mortem inspections for accuracy.
Key Gas Method (KGM) (2022) [45]	Interprets fault gases based on individual gas presence, identifying key gases for fault detection.	Concentration of key gases like C_2_H_2_ indicating fault severity.	Threshold values for C_2_H_2_ and other key gases indicative of fault type.	Relies on gas presence rather than ratios; may overlook mixed fault types.
Duval Pentagon Approach with CNN (2022) [46]	Uses all five hydrocarbon gases for fault identification, including H_2_ and C_2_H_6_, enhancing analysis for partial discharge and low energy thermal faults.	Calculates relative percentages of H_2_, C_2_H_6_, CH_4_, C_2_H_4_, and C_2_H_2_ to predict fault types.	Achieves 100 % validation accuracy in MNN model, improving fault diagnosis efficiency.	Relies on published fault databases and transformer samples, potentially biased towards specific fault scenarios.
IEC-DGA Ratio Method (2022) [48]	Uses CH_4_/H_2_, C_2_H_4_/C_2_H_6_, and C_2_H_2_/C_2_H_4_ gas ratios for fault identification.	Quantifies gas ratios (e.g., <0.1 for PD, 0.1–1.0 for D2 faults).	Provides specific threshold ranges for fault severity (e.g., >2 for C_2_H_4_/C_2_H_6_ in D2 faults).	Relies on fuzzy logic-based Simulink model and graphical rule viewer.
Using Duval Pentagon 2 (2021) [47]	Identification of gassing patterns in transformers, distinguishing between zones S, PD, and O.	Quantification of gas levels: C_2_H_6_ (up to 112 ppm), CH_4_ (up to 40 ppm), H_2_ (up to 11,500 ppm).	Average of 4 analyses per syringe; levels below 550 ppm for normal operations.	Relies on laboratory tests and visual inspections for pattern recognition.

In [48], IEC-DGA ratio method is a significant approach in transformer fault diagnosis, employing gas ratios such as CH_4_/H_2_, C_2_H_4_/C_2_H_6_, and C_2_H_2_/C_2_H_4_ to identify various fault types. It quantifies these ratios with specific metrics, distinguishing between different fault severities (e.g., <0.1 for partial discharge faults, 0.1–1.0 for higher-energy faults like D2). The method utilizes a fuzzy logic-based Simulink model and graphical rule viewer, enhancing its analytical capabilities. However, its reliance on these tools introduces potential biases, highlighting the need for expert interpretation alongside automated analysis for accurate fault diagnosis and transformer maintenance decisions.

About automatic learning, multi-method random forest (RF) machine learning model integrated with conventional dissolved gas analysis (DGA) methods. Initially, an RF model was developed and tested against individual conventional methods (DPM and DTM), showing promising but not optimal accuracy [49]. Subsequently, the model was refined by integrating five interpretation methods and comparing its performance with SVM, kNN, and Naive Bayes algorithms.

The RF model consistently outperformed these alternatives, achieving an accuracy of 92.57 %. Further optimization of RF parameters (n_estimators, max_depth, min_samples_split) through extensive testing improved accuracy and runtime efficiency. The RF multi-method approach demonstrated superior accuracy (96 %) and consistency (93.4 %) for identifying three fault types compared to conventional methods. For six fault types, it maintained strong performance with an average accuracy of 88.6 % and consistency of 87.4 %, surpassing conventional methods in both scenarios. Validation using practical transformer fault data confirmed the model's robustness and potential as a reliable tool for transformer fault identification. In Ref. [49] underscores the effectiveness of RF multi-methods in enhancing DGA interpretation, advocating for continued data enrichment to sustain and improve model performance over time.

## 4 Discussion

Dissolved Gas Analysis (DGA) as a diagnostic tool to assess transformer health by analyzing gases dissolved in insulating oil. The dataset comprised 475 records across six fault types caused by five gases: H_2_, CH_4_, C_2_H_6_, C_2_H_4_, and C_2_H_2_. The contribution of [51] centered on enhancing diagnostic accuracy through data preprocessing and algorithmic implementation. Initially, the dataset exhibited imbalance and outliers, mitigated through data filtering and the Synthetic Minority Oversampling Technique (SMOTE). This resulted in a balanced dataset of 540 records, facilitating robust model training and evaluation. We employed a Deep Neural Network (DNN), optimized via Bayesian Optimization, achieving superior performance metrics: accuracy of 98.22 %, precision of 90 %, recall of 92.5 %, and an F1 score of 94.5 %. These results underscored the efficacy of CDGA algorithm in transformer fault prediction, outperforming traditional classifiers like K-NN, Decision Tree, Random Forest, Gaussian Naïve Bayes, and SVM. The study concludes that integrating SMOTE with DNN enhances diagnostic capabilities, ensuring reliable transformer maintenance strategies in power systems. Future research avenues include refining methodology and extending applicability across diverse power industry domains.

**Fig. 9:**
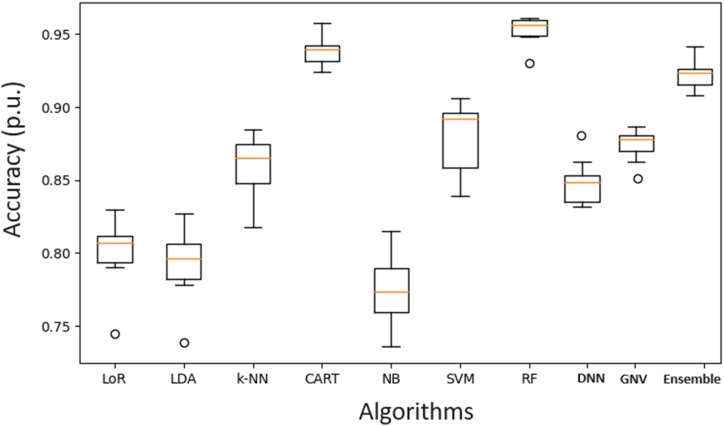
Accuracy for the model in the new database.

In Fig. 9, DGA for transformer fault prediction, the accuracy metrics revealed notable differences in performance. Logistic Regression (LoR) achieved an accuracy of 80.11 % with a standard deviation of 2.24 %, while Linear Discriminant Analysis (LDA) reached 79.23 % with a standard deviation of 2.26 %. The k-Nearest Neighbors (k-NN) algorithm demonstrated a higher accuracy of 85.94 % with a standard deviation of 2.01 %. The Classification and Regression Trees (CART) algorithm significantly outperformed these models, achieving an accuracy of 93.81 % with a lower standard deviation of 0.96 %. Naive Bayes (NB) showed the lowest accuracy at 77.44 % with a standard deviation of 2.45 %. Support Vector Machine (SVM) and Random Forest (RF) also performed well, with SVM reaching 88.00 % (2.28 %), DNN with 85 %, GNV with 88 %, ensembled algorithm (Optimal Independent Feature Selection (OIFS) method, it demonstrated superior performance, in Matlab program implementing OIFS on a dataset with 465 features completes in 16.7 s, much faster than previous methods and in Python for 3929 requires 7211s; therefore, it makes it suitable for large datasets [53].) with 92.1 %, and RF achieving the highest accuracy of 95.29 % with the lowest standard deviation of 0.89 %. These results underscore the superior performance of the RF and CART algorithms in accurately diagnosing transformer faults based on DGA data.

For ensembled method, in Ref. [53] compares the performance of the IVEL (Integrated Voting Ensemble Learning) method with other ensemble methods (RF, GBDT, LightGBM) using the feature combination from OIFS. IVEL shows higher accuracy and reduced bias, as reflected in the smaller differences in precision, recall, and F1-scores across classes compared to the other methods.

Fig. 9, to enhance the classification performance of dissolved gas analysis (DGA) in transformers, especially in the context of wind turbine transformers, several strategies can be adopted based on the latest advancements and recommendations in the field. First, data preprocessing is critical. The dataset should undergo thorough cleaning to remove outliers and handle class imbalances, which can significantly improve model accuracy. Implementing the Synthetic Minority Oversampling Technique (SMOTE) has proven effective in addressing class imbalances, as evidenced by its successful application in various studies. This approach ensures a balanced dataset, which is crucial for training robust machine learning models.

Secondly, leveraging advanced machine learning algorithms can further enhance performance. Deep Neural Networks (DNN), optimized through techniques like Bayesian Optimization, have demonstrated superior accuracy compared to traditional classifiers such as k-Nearest Neighbors (k-NN), Decision Trees, and Random Forests. The implementation of ensemble methods, such as the Integrated Voting Ensemble Learning (IVEL) method, can also improve accuracy by combining the strengths of multiple algorithms. IVEL has shown to reduce bias and increase precision, recall, and F1-scores, making it particularly effective for complex fault diagnosis scenarios.

Additionally, the use of graphical and statistical methods to analyze gas concentration trends over time is essential. For instance, IEEE standards provide specific thresholds for gas levels, which can serve as benchmarks for fault detection. Revising these thresholds, as suggested in recent research, can lead to better early fault detection and improved transformer reliability. Incorporating these thresholds into predictive models can enhance their diagnostic capabilities, particularly in identifying early-stage faults such as partial discharges and low-energy arcing, which are critical in wind turbine transformers.

Moreover, ongoing monitoring and validation of gas levels through real-time data analysis can further refine the accuracy of fault predictions. Techniques such as the Multi-Dimensional Continuity Detection and Clustering (MCDC) algorithm have shown promise in outperforming traditional models like Hidden Markov Models (HMM) and Deep Belief Networks (DBN) in terms of diagnostic accuracy.

Lastly, practical engineering case analyses should be integrated into model validation to ensure that the proposed methodologies are effective in real-world scenarios. For example, sudden faults in high-voltage transformers, which can lead to significant operational disruptions, should be analyzed to validate the predictive models' effectiveness in early fault detection.

In our analysis of Dissolved Gas Analysis (DGA) for transformer fault diagnosis, we compared various gas concentration thresholds and their implications for predictive accuracy. The IEEE standard stipulates specific limits for daily gasification: hydrogen (H_2_) at 80 ppm, methane (CH_4_) at 90 ppm, ethane (C_2_H_6_) at 90 ppm, ethylene (C_2_H_4_) at 50 ppm, acetylene (C_2_H_2_) at 1 ppm, carbon monoxide (CO) at 900 ppm, and carbon dioxide (CO_2_) at 9000 ppm. These benchmarks are essential for identifying potential faults. Our new proposal suggests revised thresholds of 100 ppm for H_2_, 250 ppm for CH_4_, 300 ppm for C_2_H_6_, 50 ppm for C_2_H_4_, 5 ppm for C_2_H_2_, 1000 ppm for CO, and 10000 ppm for CO_2_. When applying these revised limits, the accuracy of fault prediction significantly improves, enhancing the early detection of transformer issues.

About Table 4, H_2_ gas is a critical indicator in the condition monitoring of wind turbine transformers, particularly in the context of dissolved gas analysis (DGA). The presence and concentration of H₂ gas in transformer oil can signal the early stages of electrical faults, such as partial discharges or low-energy arcing. These faults can degrade the insulating properties of the transformer oil, leading to more severe issues if not detected early.

DGA methods, including those discussed in recent research, emphasize the role of H_2_ as a key diagnostic parameter. For instance, the IEC-DGA ratio method utilizes gas ratios involving H_2_ (e.g., CH_4_/H_2_) to differentiate between fault types and severities, offering a quantifiable approach to monitoring transformer health. The inclusion of H_2_ in machine learning models, such as Random Forest and CNN-based approaches, further enhances the accuracy and reliability of predictive maintenance strategies.

By carefully monitoring H_2_ levels, operators can detect and address potential faults before they escalate, ensuring the continued reliability and efficiency of wind turbine transformers. This not only reduces maintenance costs but also contributes to the overall sustainability and performance of renewable energy systems.

In reference to IEEE C57.104–2019 [5], the relative proportions of fault gases generated by partial discharges in ester fluids, as analyzed using the Duval triangle method [39] and pentagons [40], closely resemble those produced in mineral oils [41]. Under identical conditions, the quantity of fault gases measured is comparable between mineral oil and synthetic ester liquids.

To further elucidate this relationship, if the volumes of liquid and the fault conditions were identical, the concentration of a fault gas (measured in ppm) would be similar in both mineral oil and synthetic ester liquids.

This adjustment aims to provide more precise diagnostics, reducing the risk of severe transformer faults and ensuring better maintenance planning. The proposed thresholds before faults occur, notably higher for some gases (e.g., 55000 ppm for H_2_ and 1500 ppm for CH_4_), are designed to accommodate the initial warning signs of fault conditions, leading to more proactive maintenance and improved transformer reliability in Table 8.

**Table 8:** Discussion for limits.

Elements	IEEE [5] (ppm)	Limits for daily gasification (ppm/day)	New proposal (ppm)	Limits before faults proposed (ppm)
Hydrogen (H_2_)	80	100.00	100	55000
Methane (CH_4_)	90	16.00	250	1500
Ethane (C_2_H_6_)	90	–	300	300
Ethylene (C_2_H_4_)	50		50	50
Acetylene (C_2_H_2_)	1		5	5
Carbon monoxide (CO)	900		1000	1000
Carbon Dioxide (CO_2_)	9000		10000	–

Compared with [52] Hidden Markov Model (HMM), Gate Recurrent Unit (GRU), and Deep Belief Network (DBN). These algorithms are evaluated against the newly proposed Multi-Dimensional Continuity Detection and Clustering (MCDC) algorithm. The HMM uses a transition probability matrix to model sequences, GRU is a type of recurrent neural network that employs reset and update gates to learn time series transitions, and DBN is a deep learning technique that leverages multi-layer Restricted Boltzmann Machines to extract features. The metrics allow to the MCDC outperforms the others, achieving an accuracy of 0.971, macro precision of 0.970, macro recall of 0.965, and macro F1-score of 0.967, indicating superior diagnostic capability. In comparison, HMM achieves an accuracy of 0.954, GRU 0.941, and DBN 0.879.

**Fig. 10:**
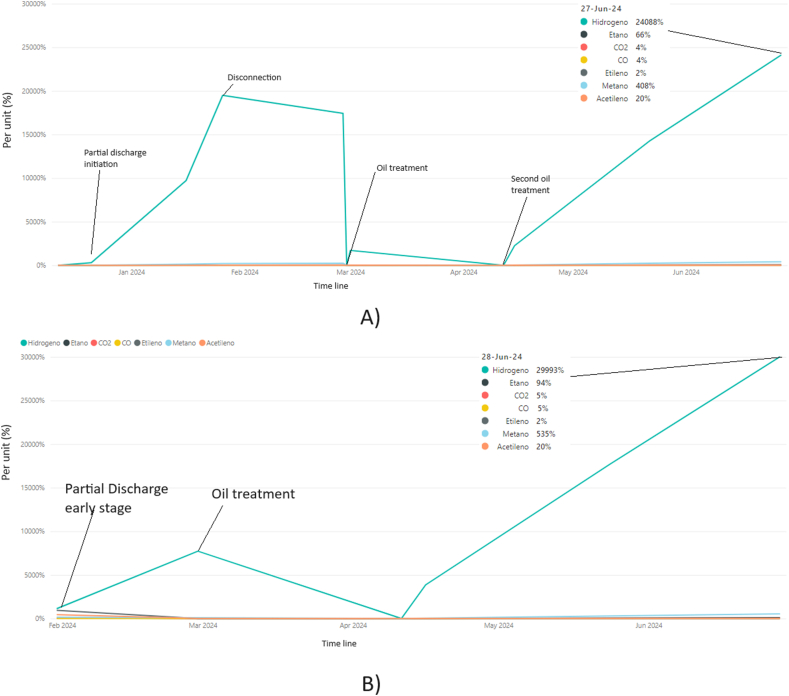
Validation for the rebound effect for partial discharge evaluation. A) First evaluation in Laboratory (Faults in terminal between cable and bushing). B) Second evaluation for partial discharge (incorrect oil filled).

From the database, the validation of the rebound effect is validated with a sudden partial discharge, in both cases, the partial discharge is confirmed for two effects: Incorrect oil installation and bushing fault based on connection between the medium voltage cable and the bushing, in Fig. 10 A), in both cases 10 days before sudden fault with hydrogen of 55000 ppm in both cases, and with methane with1500 ppm.

The analysis of the dissolved gas data from the wind turbine reveals a detailed progression of gas levels over several samples Fig. 10 A), including two repair processes related to oil treatment. The initial readings, marked as "Pass," indicate normal conditions with hydrogen (H_2_), methane (CH_4_), and other gases well within acceptable limits. Subsequent samples, however, show significant spikes in gas levels, particularly hydrogen and methane, suggesting partial discharges and arcing within the transformer. For instance, one sample shows a drastic increase in hydrogen to 19493 ppm and methane to 530 ppm, accompanied by partial discharge diagnosis and recommendations for internal inspection. Continued monitoring reveals additional samples with heightened gas levels, necessitating various recommended actions like oil changes and inspections to address the deterioration in oil properties and potential electrical issues. Oil treatment processes have shown effectiveness in reducing gas levels, as evidenced by readings taken post-treatment that return to normal or near-normal levels. For instance, after oil treatment, the hydrogen level dropped to 0 ppm, methane to 2.6 ppm, and other gases also saw significant reductions. The diagnoses post-treatment revert to "Normal," and the recommended actions focus on regular oil treatment to maintain these conditions. Throughout the process, different diagnostic methods, including Laborelec, Duval IEC 60599, and IEC 60599 Modified, consistently identify the presence of partial discharges and arcing, reinforcing the need for the proposed maintenance actions.

In the daily gas generation for Fig. 10 A), the hydrogen generated 364 ppm/day and methane 16.0 ppm/day, besides, Fig. 10 B), the hydrogen generated 361 ppm/day and methane 16.7 ppm/day,

Finally, by using the last 27 graphical methods, to two real DGA results, demonstrating their practical use, it also faces limitations related to the scope of its comparisons, the deterministic nature of the methods, and the challenges in implementing and developing new AI-based approaches [54].

In the practical engineering case analysis with [55], the occurrence of a sudden fault on April 5, in a 220 kV transformer led to significant operational issues, including the generator tripping. The fault was caused by the rupture of the high-voltage side of phase B bushing, oil leakage, deformation of the oil tank, and cracking at the lifting seat of the reinforcing rib. During the core hanging inspection on May 5, 2023, further damage was observed, including deformation and breakage of the winding in the high-voltage coil, discharge marks, and severe displacement of the iron yoke cushion block. The disassembly inspection revealed that the root cause of the malfunction was the presence of polar substances between the coil turns, leading to partial discharge and an internal short circuit. Chromatographic analysis before the fault indicated a sudden increase in methane and ethylene, which aligned with the fault occurrence; the performance of the CWGAN-GP model was compared with four other data expansion methods. The comparison highlighted the superior accuracy and effectiveness of the proposed method in diagnosing transformer faults with an accuracy of 88 % database and 92 % for the new methodology proposed.

## 5 Conclusion

The comprehensive analysis of Dissolved Gas Analysis (DGA) in transformer diagnostics underscores significant advancements in fault identification and predictive maintenance accuracy. The integration of probabilistic machine learning models, including Generative Adversarial Networks (GANs) and fuzzy logic-based approaches, has shown potential in predicting transformer lifetimes and fault conditions with high precision. However, further validation across diverse operational environments is essential to ensure their robustness.

This research article highlights the critical role of hydrogen (H₂) and other gases, such as methane and carbon monoxide, as key diagnostic parameters in transformer health monitoring, particularly within wind park transformers. With a database of 4810 DGA samples from wind parks, we observed varying concentrations and correlations among gases, revealing distinct patterns associated with different types of faults, such as partial discharges and thermal faults.

The exploration of alternative transformer oils, like palm fatty acid ester (PFAE) and virgin coconut oil, emphasizes the growing focus on environmental sustainability. While these oils demonstrate superior dielectric properties and environmental benefits, challenges related to cost-effectiveness and operational stability under varying climatic conditions remain.

Advancements in predictive maintenance methodologies, particularly the use of advanced machine learning models like Random Forest (RF) and Integrated Voting Ensemble Learning (IVEL), have enhanced the accuracy of fault diagnosis. These methods, combined with data preprocessing techniques such as the Synthetic Minority Oversampling Technique (SMOTE), have proven effective in addressing class imbalances and improving model performance.

Furthermore, the adoption of novel sensor-oriented approaches for mechanical state recognition, as well as emerging technologies like Operational Modal Analysis and multisource data fusion, offers promising solutions for transformer diagnostics. However, the practical implementation of these technologies requires a critical evaluation of their feasibility in real-world scenarios.

Overall, this study underscores the need for continued research and innovation in transformer diagnostics and oil insulation technologies. The findings support the proposal of revised gas concentration thresholds, which significantly improve fault prediction accuracy and enable more proactive maintenance strategies. Continued validation of these methods, particularly in wind turbine transformers, is crucial for ensuring the reliability and sustainability of power systems in the face of evolving energy demands. Based on the comprehensive analysis of dissolved gas analysis (DGA) in transformer diagnostics and the advancements in predictive maintenance methodologies, it is evident that significant progress has been made in enhancing fault identification and prediction accuracy. The integration of probabilistic machine learning models, such as Generative Adversarial Networks (GANs) and fuzzy logic-based approaches, has demonstrated promising results in predicting transformer lifetimes and fault conditions with high precision. However, these methodologies require further validation and adaptation to ensure robust performance across diverse operational environments.

Furthermore, the exploration of alternative transformer oils, including biodegradable options like palm fatty acid ester (PFAE) and virgin coconut oil, highlights a growing emphasis on environmental sustainability in power system applications. While these oils offer superior dielectric properties and environmental benefits, challenges such as cost-effectiveness and operational stability in varying climatic conditions remain critical considerations for widespread adoption. Overall, the findings underscore the need for continued research and innovation in transformer diagnostics and oil insulation technologies to meet evolving energy demands sustainably and reliably. Based on the systematic review with PRISMA-A, a large database with oil samples composed of 3929 for training and 829 for test was identified to evaluate the machine learning techniques; with the major accuracy by using RF compared with entropy, confusion metrics and entropy.

## 6 Future works

For future research in transformer health monitoring and fault prediction: Investigate advanced methodologies integrating AI and machine learning, focusing on deep learning models for accurate fault prediction. Implement real-time monitoring through IoT devices to enable proactive maintenance strategies and reduce downtime. Develop enhanced interpretation methods for Dissolved Gas Analysis (DGA) data, integrating multiple data sources for improved diagnostic accuracy. Explore nanotechnology applications for transformer materials to enhance reliability under dynamic conditions. Assess the environmental impact of insulation materials and promote biodegradable alternatives. Standardize fault detection techniques, validate new methodologies through field trials, and establish performance metrics. Address cybersecurity concerns in IoT systems and develop cost-effective retrofitting strategies. Design intuitive interfaces and decision support systems integrating AI analytics for informed maintenance decisions. Foster cross-disciplinary collaboration to advance transformer health monitoring and sustainability in the power industry.

Future endeavors should focus on integrating advanced analytics with real-time monitoring capabilities to optimize asset management and ensure resilient performance in renewable energy infrastructure.

Based on the results of the review, the authors suggest several implications for practice, policy, and future research in the field of transformer fault diagnostics:

Practice.

• Enhanced Diagnostic Accuracy: The integration of advanced machine learning models, such as Generative Adversarial Networks (GANs) and fuzzy logic-based approaches, has demonstrated significant improvements in predicting transformer lifetimes and fault conditions. Practitioners should consider adopting these models to enhance fault identification and predictive maintenance accuracy.
• Adoption of Alternative Transformer Oils: The study highlights the benefits of using biodegradable transformer oils like palm fatty acid ester (PFAE) and virgin coconut oil. While these oils offer environmental advantages and superior dielectric properties, their cost-effectiveness and operational stability need to be addressed. Practitioners should evaluate these factors when considering alternative oils for transformers, particularly in varying climatic conditions.
• Revised Gas Concentration Thresholds: The findings support the proposal for updated gas concentration thresholds in transformer diagnostics. Implementing these revised thresholds can significantly improve fault prediction accuracy and facilitate more proactive maintenance strategies.

Policy.

• Environmental Sustainability: The growing emphasis on environmental sustainability in transformer oil selection should inform policy decisions. Policymakers may consider promoting the use of biodegradable oils through incentives or regulations to support environmentally friendly practices in power systems.
• Standards and Guidelines: The advancements in predictive maintenance methodologies and novel sensor-oriented approaches should lead to the development of updated standards and guidelines for transformer diagnostics. Policymakers and industry standards organizations should incorporate the latest research findings into regulatory frameworks to ensure the adoption of best practices.

Future Research.

• Validation of Advanced Models: Further research is needed to validate the performance of advanced machine learning models across diverse operational environments. This includes testing models like GANs and fuzzy logic-based approaches in various settings to ensure their robustness and reliability.
• Operational Stability of Alternative Oils: Future studies should focus on the operational stability of alternative transformer oils under different climatic conditions. Research should address the challenges related to cost-effectiveness and performance to facilitate broader adoption of environmentally friendly oils.
• Feasibility of Emerging Technologies: The practical implementation of emerging technologies, such as Operational Modal Analysis and multisource data fusion, requires further investigation. Research should evaluate the feasibility and effectiveness of these technologies in real-world transformer diagnostics scenarios.

## Funding

No funding.

## Declaration of competing interest

The authors declare that they have no known competing financial interests or personal relationships that could have appeared to influence the work reported in this paper.
